# Fungal and bacterial successions in the process of co-composting of organic wastes as revealed by 454 pyrosequencing

**DOI:** 10.1371/journal.pone.0186051

**Published:** 2017-10-23

**Authors:** Polina Galitskaya, Liliya Biktasheva, Anatoly Saveliev, Tatiana Grigoryeva, Eugenia Boulygina, Svetlana Selivanovskaya

**Affiliations:** 1 Department of Applied Ecology, Institute of Environmental Sciences, Kazan Federal University, Kazan, Russian Federation; 2 Department of Ecological Systems Modeling, Institute of Environmental Sciences, Kazan Federal University, Kazan, Russian Federation; 3 Institute of Fundamental Medicine and Biology, Kazan Federal University, Kazan, Russian Federation; Estacion Experimental del Zaidin - CSIC, SPAIN

## Abstract

Composting is viewed as one of the primary methods to treat organic wastes. Co-composting may improve the efficiency of this treatment by establishing the most suitable conditions for decomposers than those present in the individual wastes. Given that bacteria and fungi are the driving agents of composting, information about the composition of their communities and dynamics during composting may improve reproducibility, performance and quality of the final compost as well as help to evaluate the potential human health risk and the choice of the most appropriate application procedure. In this study, the co-composting of mixtures containing two similar components (organic fraction of municipal solid waste and sawdust polluted by oil) and one discriminate component (sewage sludges of different origin) were investigated. Bacterial and fungal community successions in the two mixtures were analyzed during the composting process by determining the change in their structural dynamics using *q*PCR and 454 pyrosequencing methods in a lab experiment for a period of 270 days. During the initial composting stage, the number of 16S bacterial copies was (3.0±0.2) x 10^6^ and (0.4±0.0) x 10^7^ g^-1^, and the *Rhodospiralles* and *Lactobacialles* orders dominated. Fungal communities had (2.9±0.0) x10^5^ and (6.1±0.2) x10^5^ ITS copies g^-1^, and the *Saccharomycetales* order dominated. At the end of the thermophilic stage on the 30^th^ day of composting, bacterial and fungal communities underwent significant changes: dominants changed and their relative abundance decreased. Typical compost residents included *Flavobacteriales*, *Chitinophagaceae* and *Bacterioidetes* for bacteria and *Microascaceae*, *Dothideomycetes*, *Eurotiomycetes*, *Sordariomycetes*, and *Agaricomycetes* for fungi. During the later composting stages, the dominating taxa of both bacterial and fungal communities remained, while their relative abundance decreased. In accordance with the change in the dominating OTUs, it was concluded that the dynamics of the bacterial and fungal communities were not similar. Analysis by non-metric multidimensional scaling (NMDS) revealed that the bacterial communities of the two composts became progressively more similar; a similar trend was followed by the fungal community.

## Introduction

Accelerated by exponential population growth and increased consumption per capita, organic wastes have become a serious environmental problem in the past decades [1]. Organic wastes are biodegradable, thus, they can be treated with appropriate energy and resources. The natural process of aerobic decomposition of organic matter can be used as the basis for the controlled treatment of waste in a process referred to as composting. Composting has been successfully used as an organic waste reduction tool worldwide [2].

The process of composting has been widely studied and described. Interest in compost microbiota has increased significantly due to the fact that bacteria and fungi are the two main driving forces of composting, and efficient composting is dependent on the presence of a high microbial diversity [3–7]. The methods previously used (culture-dependent or fingerprinting methods) did not allow for an accurate determination of microbial successions during composting. New sequencing methods now permit researchers to obtain information on the level of both dominant and minor species, at the family, genera or individual species level. It is necessary to add that very few publications analyze bacterial and fungal communities of the composts simultaneously [8–10]. Understanding the changes in microbial communities during composting may improve reproducibility, performance, quality of the final compost as well as the evaluation of potential human health risks and choice of the optimal application procedure [11–13].

The decomposition of organic matter by composting is usually divided into four main stages that differ in physico-chemical conditions such as temperature–mesophilic, thermophilic, cooling and curing/maturation [3,4,14,15]. We suggest that during these stages, microbial succession takes place, and strains that decompose increasingly recalcitrant organic matter are selected [3,8,16].

In some cases, a single waste type is not suitable for composting because it may have high concentrations of one type of nutrient, low or high pH or other factors. Co-treatment of organic wastes with compensative properties can be more efficient in terms of time, need of fertilizers or quality of the final product [17–20]. Analysis of the interaction of different organic materials as a source of their own microbial community may help in understanding the microbial successions and to increase the effectiveness of the composting process. There are different opinions concerning microbial community formation in composts. One theory states that a microbial community is influenced more by physical location and conditions of composting [21], while another theory associates the presence and absence of microbes with temperature fluctuations [8,11,16,22]. Supporters of a third theory believe that microbial composition is determined by the initial properties and microbiota of the composting material. Therefore, the final compost is re-colonized by the organisms abundant during the mesophilic stage and those able to survive the thermophilic stage in the form of spores [23]. De Gannes and colleagues (2013) suggest that each composting microbial community is a unique system, and no relationship exists between different composts in terms of community composition [22].

We hypothesize that the bacterial and fungal communities of two different compost mixtures at the final stage of composting are more similar than that in the initial composts, and that a one-direction succession occurs without recolonization of the final compost by species from the mesophilic stage. We also hypothesize that shifts in bacterial and fungal composting communities do not occur simultaneously. Therefore, the bacterial and fungal communities were analyzed during the composting of two mixtures containing either an organic fraction of municipal solid waste (MSW) or sawdust polluted by oil. The third component of both mixtures was sewage sludge (SS), which was obtained from two different waste water treatment plants. The first plant treats water from a factory where organic compounds are synthesized, and the second plant treats mixed municipal household and industrial waste water in a city with a population of half a million.

## Materials and methods

### Compost mixtures

Two compost mixtures were prepared using two identical components–organic fraction of MSW and sawdust polluted by oil–and one discriminate component–wet industrial sewage sludge (I compost) or dewatered sludge of mixed industrial and household waste water (IH compost). The proportion of the components was calculated in order to reach the optimal moisture content (between 55% and 65%) and C:N ratio (from 25 to 30) [24,25]. The organic fraction of MSW, sawdust polluted by oil, and two different SS samples were mixed in the following proportions: 2.7:3:1 for I compost and 1:1:3 for IH compost weight/weight on fresh weight basis (w/w). The chemical characteristics of the raw wastes and composting mixtures are presented in Table 1.

**Table 1:** The chemical characteristics of the raw wastes and composting mixtures.

Parameter	Industrial SS	Industrial household dewatered SS	Organic fraction of MSW	Sawdust polluted by oil	I compost, day 0	IH compost, Day 0
**pH**	7.2±0.8	6.0±0.6	4.4±0.5	6.3±0.7	6.1±0.8	5.9±0.6
**Moisture, %**	99.0±11.2	63.0±5.8	72.0±6.9	6.0±0.6	58.0±5.6	62.0±4.7
**Total N, %**	4.0±0.4	2.2±0.2	1.8±0.0	1.4±0.1	1.0±0.0	1.1±0.0
**Organic carbon, %**	25.0±1.6	11.3±1.1	19.0±1.2	67.8±5.7	31.5±2.6	27.9±3.1
**Metals:**	n.d.*	n.d.*	n.d.*	n.d.*		
**Al mg kg**^**-1**^					357.9±39.4	2942.8±202.5
**Cd mg kg**^**-1**^					<0.05	<0.05
**Co mg kg**^**-1**^					10.5±1.2	4.2±0.3
**Cr mg kg**^**-1**^					7.1±0.6	37.5±0.4
**Cu mg kg**^**-1**^					40.3±3.7	20.5±0.2
**Fe mg kg**^**-1**^					3502.7±347.6	4421.6±360.7
**Mn mg kg**^**-1**^					42.6±3.8	112.4±14.3
**Ni mg kg**^**-1**^					3.5±0.4	10.5±1.4
**Pb mg kg**^**-1**^					2.8±0.1	7.5±0.6
**Zn mg kg**^**-1**^					130.3±12.0	204.8±21.1

*no determination was conducted

After preparation, the mixtures were divided into three parts, and each part was considered as an independent replication and analyzed separately. The weight of each replication of initial compost was approximately 20 kg.

The moisture content of the compost was maintained at a level of 55–60%. Composts were incubated for 270 days at room temperature and aerated by mixing daily. Ten samples were randomly sampled from different regions of the compost piles and merged together to obtain a composite sample. The procedure was repeated three times for each compost replication. As a result, nine samples of each compost mixture type were analyzed at each sampling time. Samples were immediately used for chemical analysis or DNA extraction.

### Estimation of dissolved organic carbon (DOC) content, pH and temperature of compost samples

The pH was analyzed in a 1:10 (w/v) water extract. The temperature was measured using a standard lab mercury thermometer. The DOC was extracted from the compost samples using a 0.5 M K_2_SO_4_ solution (*V*/*W* = 1:5) by shaking for 30 min, followed by centrifugation for 10 min (2880 g). In addition, the organic carbon content was estimated according to ISO 14235:1998 [26]. Phytotoxicity was estimated using oat plants (*Avena sativa*) via the contact method according to ISO 11269–1 (2012) and ISO 11269–2 (2012) [27,28]. Germination index (GI) was calculated as described by Zucconi et al. (1981) and used as a phytotoxicity parameter. GI (%) combined measurement of relative seed germination and relative root elongation [29].

### DNA extraction

Total genomic DNA of the samples was extracted from composite sample using a bead-beating procedure with a Retsch MM 400 Mixer Mill (Fisher Scientific, Thermo Fisher Scientific, Waltham, Massachusetts, USA) using FastDNA®SPIN Kit for Soil (Bio101, Qbiogene, Heidelberg, Germany) according to the manufacturer’s instruction. In all cases, DNA extractions were done in triplicate and were cleaned using DNeasy PowerClean Pro Cleanup Kit (Quiagen, Germany). The DNA samples stored at −20°C or analyzed immediately.

### Quantitative amplification

Quantitative polymerase chain reaction (qPCR) was conducted using DNA samples from nine replicates of each compost mixture. For bacteria, 16S 984f (5’-AACGCGAAGAACCTTAC-3’) and 1378r (5’-CGGTGTGTACAAGGCCCGGGAACG-3’) primers were used [30,31]. In the fungal assay, ITS1 (5’-TCCGTAGGTGAACCTGCGG-3’) and ITS2 (5’-GCTGCGTTCTTCATCGATGC-3’) primers were used [32].

PCR reactions were conducted with a 0.1 U μl^-1^ SynTaq Polymerase, 1x Buffer with SYBR Green, 2.5 mM MgCl_2_, 200 μM dNTPs each, 0.2 μM primer each and 1 μl of DNA template in the CFX96 Touch Real-Time PCR Detection System (Bio-Rad, Munich, Germany). The qPCR program consisted of initial denaturation at 95°C for 5 min followed by 39 three-step cycles of 62–60°C for 45 s, 95°C for 15 s, and 72°C for 30 s. All of qPCR assays performed with efficiency of more than 94%, R2 values greater than 0.99.

The standard curves were generated for bacteria using serial DNA dilutions of DNA of *Bacillus pumilus* and *Penicillium chrysogenum*. The concentration of bacterial and fungal DNA was measured on a Qubit 2.0 Fluorometer (Invitrogen, Thermo Fisher Scientific, Waltham, Massachusetts, USA) using the Picogreen ds DNA reagent (Invitrogen Ltd., Paisley, UK).

### 454 pyrosequencing

For pyrosequencing, DNA samples obtained from several replicates of each compost mixture at each sampling point were merged together. This is because no significant differences were found between the replicates in the preliminary experiments (S1-S6).

The ITS1 region of the fungal rRNA gene complex was amplified with the ITS4/ITS1 primers [33,34], and the bacterial V4 region of the 16S rRNA was amplified with the 27f/533r primers [35] using GS Junior Technology (Roche 454 Life Sciences, Branford, USA). Fusion primers with TCTCAGAGAGTTTGATCCTGGCTCAG barcode for bacteria and CTTGGTCATTTAGAGGAAGTAA for fungi were used.

For amplification, each 25-μl reaction mixture contained 1 μM template DNA, 0.1 U μl^-1^ HiFi Polymerase (GS Junior Titanium emPCR Kit (Lib-A)), FastStart 1x Buffer #2, 200 μM dNTPs each, of 0.2 μM of primers. Cycling conditions were: 95°C for 3 min of initial denaturation and 35 cycles of 95°C for 15 s of dissociation, 55–65°C for 45 s of annealing, 72°C for 1 min for each cycle extension and 72°C for 8 min of a final extension step.

Amplicons very purified and concentrations were measured on a Qubit 2.0 Fluorometer (Invitrogen, Thermo Fisher Scientific, Waltham, Massachusetts, USA) using the Picogreen ds DNA reagent (Invitrogen Ltd., Paisley, UK), and the libraries were pooled in equimolar concentrations to prepare for pyrosequencing. Emulsion PCR and sequencing of the pooled samples in a single 454 GS Junior run were carried out according to the manufacturer’s instructions.

### 454 data and statistical analysis

The 454-pyrosequencing data were processed by using the Quantitative Insights Into Microbial Ecology (QIIME) platform, version 1.6.0. [36].

The taxonomic classification of each phenotype was determined in accordance with the Greengenes database. Operational taxonomical units (OTUs) were clustered with a similarity cutoff at 97%. The OTU data were used to calculate the richness and diversity indices of the bacterial community and the relative abundance of phylogenetic groups in the soils. Two OTUs of fungi were identified using the Unified system for the DNA-based fungal species linked to the classification (UNITE) Ver. 7.0 [37].

The 454-sequencing data obtained were published in European Nucleotide Archive, with the accession numbers ERR1906837- ERR1906852.

Statistical analyses were performed using Origin 8.0 (OriginLab, Northampton, USA) and R Statistical Software (R 3.0.0, R Foundation for Statistical Computing Version, Vienna, Austria) packages [38]. Three indices of alpha diversity calculated for each community were the Shannon-Weaver index [39], Simpson index [40] and Shannon’s evenness [41].

Relative similarities of bacterial and fungal communities revealed in the two composts were verified using non-metric multidimensional scaling (NMDS) based on the Bray-Curtis coefficient according to a procedure presented by Clarke (1993) [42–44].

## Results and discussion

### Compost properties

**Fig 1:**
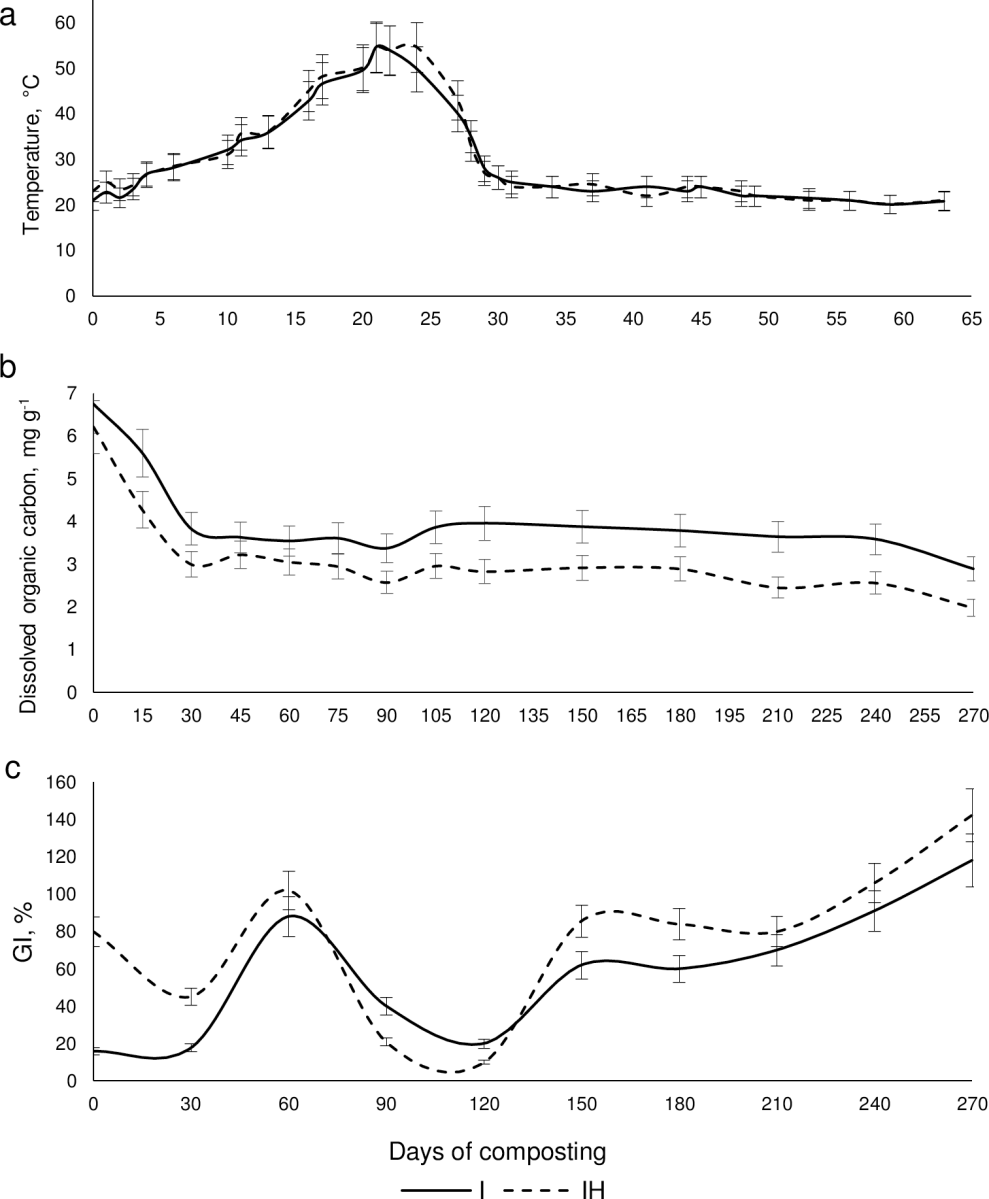
Dynamics of temperature, DOC and GI in the process of composting.

The temperature dynamics and changes in DOC and phytotoxicity of two composts are presented in Fig 1. These parameters are recommended as appropriate indicators of composting effectiveness and stage [35]. The temperature peak observed on the 20th-24th days of composting in both mixtures, the DOC level fell significantly during the first four weeks and then remained stable, and the GI reflecting the maturity of the composts exceeded 100% on the 270th day. Summarizing the results obtained for each compost we can suggest that the mesophilic stage in both composts lasted for 16 days, and the thermophilic stage lasted for approximately 12 days. Thus the cooling stage began on the 28th day for both I and IH composts. At the same time, there were no significant differences in pH level dynamics estimated in the composting process, and the pH level remained neutral throughout the entire composting process.

According to the temperature, GI and DOC changes, shifts in microbial communities were analyzed in the two composts on the 2nd day, which reflects the beginning of the process of composting, 30th day, which was the time of transition from the thermophilic to cooling stage (presumably, many thermophilic and thermo-tolerant species were abundant at this sampling point). Besides, we characterized the microbial community of the immature compost on the 150th day (which was in the middle of the experiment), and that of the final mature compost was characterized on the 270th day.

### Relative abundance of bacteria and fungi as revealed using quantitative PCR

Results calculated from qPCR standard curves are shown in Table 2 [22]. In the I compost, the number of bacterial 16S and fungal ITS copies ranged between (3.0±0.2) x 10^6^ and (1.1±0.0) x10^8^ and between (0.6±0.0) x 10^5^ and (2.9±0.0) x 10^5^copies per gram of dry compost, respectively. The number of copies in the IH composts ranged between (0.4±0.0) x 10^7^ and (4.9±0.5) x 10^7^ copies g^-1^ for bacteria and between (0.9±0.0) x 10^5^ and (6.1±0.2) x 10^5^ g^-1^copies for fungi. In general, the number of bacterial copies was more than those of fungi by 7–846 times. The excess of bacterial copies as compared with fungal copies has been previously observed [45].

**Table 2:** Abundance of bacteria and fungi in composts of different ages as revealed by qPCR.

Day of composting	Number of copies of microbes in I samples, g ^- 1^ dry compost		Number of copies of microbes in IH samples, g ^- 1^ dry compost	
	Bacteria	Fungi	Bacteria	Fungi
**2**	(3.0±0.2) 10^6^	(2.9±0.0) 10^5^	(0.4±0.0) 10^7^	(6.1±0.2) 10^5^
**30**	(8.0±0.3) 10^6^	(0.6±0.0) 10^5^	(2.0±0.1) 10^7^	(0.9±0.0) 10^5^
**150**	(1.1±0.0) 10^8^	(1.3±0.0) 10^5^	(4.9±0.5) 10^7^	(2.5±0.0) 10^5^
**270**	(2.6±0.8) 10^7^	(1.8±0.0) 10^5^	(1.8±0.0) 10^7^	(1.2± 0.0) 10^5^

In both compost samples, minimal levels of fungal ITS copies were observed on the 30^th^ day of composting. This finding is consistent with previous results showing a decrease in the amount of fungi during thermophilic composting stages and a subsequent increase during cooling [8]. The decrease can be explained by the fact that compost fungi are not thermo-tolerant, in contrast to bacteria.

It must be noted that the dynamics of bacterial and fungal copy numbers were not equal within one and the same compost, while bacteria of two different composts fluctuated in numbers similarly, as did fungi. We suggest, that this can explain by the differences of preferred substrates: bacterial numbers grow when easily degradable organic compounds are available, and fungal numbers grow when recalcitrant compounds such as lignin or cellulose are intensively decomposed [8,11,22].

### Bacterial community composition

After data denoising and chimera screening (12,939 chimera were excluded from the analysis), a total of 62,186 sequences were used for further identification. Approximately 20,049 sequences (26.7%) could be identified at the genus level. In total, 356 unique bacterial OTUs were found in compost materials using 454 pyrosequencing. The lowest number of OTUs (52) was observed in I compost samples at the beginning of the process. The average number of OTUs was lower in the I samples (107) than the IH samples (150). The maximum number of OTUs (157) was observed in the IH compost on the 270^th^ day of incubation. No correlation was found between the duration of composting and OTU numbers in the samples.

The highest number of OTUs belonged to *Proteobacteria* (mainly, *Alpha* and *Gamma*) and *Firmicutes* phyla. This is consistent with data presented previously [18,45–47]. The abundance of *Alphaproteobacteria* remained consistently high throughout the whole process of composting in both compost types. Interestingly, it was twice as high in the I composts as compared with IH composts. The abundance of *Gammaprotebacteria* increased with time, while the most drastic increase occurred during the first month of composting. Similarly, the abundance of *Firmicutes* fell dramatically from the 2^nd^ to the 30^th^ day composting, and then remained quite low.

#### Bacterial community composition of the I compost. Initial stage of composting

The 20 most abundant bacterial taxa in the I and IH composts are presented in Table 3. *Rhodospiralles* and *Lactobacialles* were the two dominating orders in the I compost on the 2^nd^ day of incubation with 64.9% and 31.7% relative abundance, respectively. Within the *Rhodospiralles* order, two OTUs played the main role: *Gluconobacter* with 47.7% and the *Acetobacteraceae* family with 64.8%. *Gluconobacter* strains were reported to be typical inhabitants of sugary niches such as apples, dates, garden soil, baker's soil, fruits etc. [48]. *Gluconobacter* could have originated from the organic fraction of MSW-containing food remains including fruits and vegetables that were used for preparation of the I compost mixture.

**Table 3:** The most abundant bacterial OTUs found in I and IH composts.

Phylum	Class	Order	The most abundant OTUs/taxa (if applicable)	Relative bacterial abundance in composts of different ages, %							
				I composts				IH composts			
				days of composting							
				2	30	150	270	2	30	150	270
*Proteobacteria*	*Alphaproteobacteria*	*Rhizobiales*	*Parvibaculum*	0.4	33.2	33.7	23.9	3.2	8.6	15.6	7.8
*Proteobacteria*	*Gammaproteobacteria*	*Chromatiales*	*Ectothiorhodospiraceae*	0.1	11.3	11.6	27.4	0.6	7.1	20.5	10.3
*Proteobacteria*	*Alphaproteobacteria*	*Rhodospirillales*	*Gluconobacter*, *Acetobacteraceae*	64.9	1.4	0.3	2.5	12.9	0.6	0.5	5.8
*Firmicutes*	*Bacilli*	*Lactobacillales*		31.7	0.1	0.0	0.0	49.2	0.1	0.1	0.0
*Proteobacteria*	*Gammaproteobacteria*	*Xanthomonadales*	*Xanthomonadaceae*	0.6	7.5	8.3	6.6	4.9	10.2	4.7	7.1
*Bacteroidetes*	*Bacteroidia*	*Bacteroidales*		0.1	3.8	6.2	0.5	1.0	18.6	7.8	3.8
*Proteobacteria*	*Alphaproteobacteria*	*Sphingomonadales*		0.0	3.8	4.5	6.9	1.5	1.9	4.3	3.2
*Spirochaete*	*Spirochaetes*	*Sphaerochaetales*		0.0	4.0	8.7	0.2	0.0	11.1	1.2	0.3
*Proteobacteria*	*Betaproteobacteria*	*Burkholderiales*	*Comamonadaceae*	0.5	2.9	2.6	1.3	5.6	5.3	4.2	1.9
*Proteobacteria*	*Alphaproteobacteria*			0.0	0.4	0.4	8.5	0.2	0.2	4.5	6.1
*Proteobacteria*	*Alphaproteobacteria*	*Elli*		0.0	5.4	5.0	4.3	0.1	1.3	0.7	0.9
*Bacteroidetes*	*Flavobacteriia*	*Flavobacteriales*	*Flavobacterium*	0.0	2.3	1.4	0.2	0.9	4.7	3.6	4.4
*Actinobacteria*	*Actinobacteria*	*Actinomycetales*		0.4	2.1	0.7	1.4	2.6	1.7	3.0	5.7
*Proteobacteria*	*Betaproteobacteria*	*Rhodocyclales*		0.2	2.8	1.9	3.8	0.2	2.5	1.7	2.3
*Proteobacteria*	*Alphaproteobacteria*	*Caulobacterales*		0.0	1.6	1.8	1.4	2.5	1.9	0.8	0.8
*Bacteroidetes*	*[Saprospirae]*	*[Saprospirales]*	*Chitinophagaceae*	0.0	2.0	1.0	0.9	0.5	1.9	2.6	2.7
*Proteobacteria*	*Alphaproteobacteria*	*BD7-3*		0.0	1.4	1.9	2.7	0.0	0.6	3.6	0.6
*Firmicutes*	*Clostridia*	*Clostridiales*		0.0	0.9	0.8	0.3	0.7	5.8	1.7	0.7
*Proteobacteria*	*Alphaproteobacteria*	*Rhodobacterales*		0.1	2.9	1.7	0.2	0.3	0.5	1.7	0.9
*Proteobacteria*	*Betaproteobacteia*	*Hydrogenophilales*		0.0	0.0	0.0	0.0	2.6	0.2	2.0	3.0

Among *Lactobacilliales*, one OTU strongly dominated. However, it was not identified at the family or genera level. *Lactobacillus* spp. are typical residents of composts in the initial stages of the process [49]. Overall, our findings support previously published data claiming that mesophilic *Lactobacillus* spp. and *Acetobacter* spp. are the major bacterial groups in the mesophilic stage of composting [50–52].

#### Evolution of bacterial community in the process of composting

On the 30^th^ day of composting, the species dominating the bacterial communities in I composts changed: *Rhizobiales*, *Chromatiales* and *Xanthomodales* became the most abundant orders with 33.2, 11.2 and 7.6%, respectively. The taxa dominating on the 30^th^ day of composting had a high relative abundance throughout the entire composting process. Notably, the relative abundance of the dominant taxa on the 30^th^ day of composting was less than that on the 2^nd^ day.

Detailed information concerning the order *Xanthomodales* is provided above. It should be mentioned that strains belonging to this taxa have previously been found in composts [2,22].

Within the *Rhizobiales* and *Chromotiales* orders, two dominating OTUs were identified that are not typical of composts. *Rhizobiales* are known as nitrogen fixers and are found in soil and on plant roots and are also plant and animal pathogens [53]. *Rhizobiales* were found in composts previously [22]. However, *Parvibaculum* sp. was the most abundant and unexpected species in our compost analysis. *Parvibaculum* sp. was found by other researchers in many different environments polluted by hydrocarbons or other organic compounds [54]. Notably, the abundance of *Parvibaculum* sp. was very low in both initial composts. Intensive development of *Parvibaculum* sp. populations can be explained by the presence of oily polluted sawdust as one of the composts’ components. *Parvibaculum* sp. use hydrocarbons and lubricants as a carbon substrate and grow actively, but this decomposition does not occur in the polluted sawdust. It begins only after preparation of the compost mixture and optimization of conditions for hydrocarbon biodegradation.

The order *Chromatiales* was basically presented by one OTU belonging to the *Ectothiorhodospiraceae* family. This OTU was genetically related to the species found in sewage sludge and oil-polluted sites [55,56].

#### Bacterial community composition of the IH compost. Initial stage of composting

In general, the bacterial community of IH composts was more stable during the initial stages versus that in the I compost, in terms of the number of dominating and sub-dominating taxa and their abundance.

In the IH composts, *Lactobacialles* dominated with 49.2%, while *Rhodospiralles* together with *Burkholderiales* and *Xanthmonadales* were sub-dominants with 12.9, 5.6 and 4.9%, respectively, and another 17.8% was represented by orders with amounts ranging from 1 to 3.2%.

As in the I compost, the *Gluconobacter* and *Acetobacteraceae* families predominated within the *Rhodospiralles* order, however, their relative abundance was ~5-fold lower in IH composts.

The sub-dominating taxa *Xanthomodales* was represented mainly by species of the *Xanthomonadaceae* family. *Xanthomonadaceae* are aerobic, motile, catalase and oxidase positive bacteria [57] found in soil [58]. *Pseudoxanthomonas* genera with their maximum abundance on the 30^th^ and 150^th^ days of incubation were identified in composts previously by de Gannes with colleagues (2013) [22]. *Stenotrophomonas* was one of the *Xanthomonadaceae* genera identified in our analyses. This plant-pathogenic genus [59] was abundant in both composts until the 150^th^ day but not in the final product. Among *Burkholderiales* representatives of *Comamonadaceae* dominated. Bacteria belonging to this family were found in sewage sludge [60,61].

#### Evolution of bacterial community in the process of composting

As in I composts, the dominating species of the bacterial community of IH changed on the 30^th^ day of composting. Although *Rhizobiales*, *Chromatiales* and *Xanthomodales*, (which dominated in I composts) were quite abundant, the *Bacteroidales* and *Sphaerochaetales* orders played a more important role with 18.6 and 11.1%, respectively. During later stages, the relative abundance of the dominating taxa fell, and that of the other taxa rose. As a result, at the completion of the composting process, the bacterial community of the IH mixture was represented by ten strains with 3–9%, in contrast to the I mixture in which the relative abundance of the dominants remained quite high (20–25%).

*Bacteroidales* and *Xanthomodales* were described above. *Spaerochaeteatales* are able to use organic acids as carbon source, and they were rarely found in composts and sludge [62,63].

As in I composts, the two dominating OTUs within the *Rhizobiales* and *Chromotiales* orders were *Parvibaculum* sp. and the *Ectothiorhodospiraceae* family. In IH composts, the proportion of *Parvibaculum* sp. among *Rhizobiales* was calculated to be 75, 62 and 17% after 30, 150 and 270 days of composting, respectively, while *Parvibaculum* sp. proportions remained high in I composts until the completion of the process (82%). Interestingly, the mass of polluted sawdust is higher in the I compost, and the population of *Parvibaculum* sp. in this compost remained abundant until the end of the process. In contrast, in the IH compost where the mass of polluted sawdust is lower, the abundance of *Parvibaculum* sp. decreased indicating that hydrocarbons were biodegraded. Thus, the presence of hydrocarbons preconditioned the development of hydrocarbon-degrading species which out-competed the others. This species then transformed the environment which became unfavorable for its survival, and the dominant species were then substituted by new species with a greater chance of survival under the new conditions.

#### Additional remarks concerning bacterial community structure in I and IH composts

There are several explanations concerning the taxa that did not belong to dominants.

*Flavobacteriales*, *Chitinophagaceae* and other representatives of the *Bacterioidetes* phylum are able to degrade macromolecules such as chitin and cellulose. Their abundance was demonstrated to be constant in different composts [7,45,64,65]. In our experiment, with both composts, the relative abundance of these taxa increase from the 2^nd^ to the 30^th^ day, and then it remained stable or increased slowly. In all cases, the abundance of all the taxa mentioned was higher in the final composts in comparison with the initial compost mixtures. Possibly species of these taxa were more competitive in the composts due to their metabolic properties, and therefore they remained in the community even during the process of succession.

*Actinomycetes* are known as active cellulose decomposers, and as typical residents of composts [66], they are called the key players of composting [67,68]. However, *Actinomycetales* were observed in only small proportions in the investigated composts ranging from 0.37 to 5.7%.

Interestingly, a dominance of *Bacillus* was not observed in the compost samples investigated in our analyses, even though bacteria of this genus are described as predominant in the thermophilic and maturation phases and throughout the entire composting process [15,52]. However, these findings support the results of Franke-Whittle et al. (2014) who suggested that *Bacillus* sp. are outcompeted by the other bacteria in the process of co-composting of digestates [69].

The *Clostridia* class includes pathogens as well as free-living species that are anaerobic. In our investigation, 23 OTUs belonging to the class *Clostridia* were identified, and one to the order *Clostridiales* belonging to the 20 most abundant ones (Table 3). Its relative abundance was higher on the 30^th^ and 150^th^ days of incubation as compared to the 2^nd^ and 270^th^ days in both composts. The presence of *Clostridia* and their role in the decomposition of cellulose in composts has been previously described [70–72].

In general, the results of our analyses of the bacterial communities of the two composts are consistent with those of López-González et al. (2015) [73]. These researchers suggested that in the early stages of composting, the microbial composition largely depends on the presence of raw materials, and biotic and abiotic factors (such us temperature, water content, nutrient compounds) play a more important role. However, the final stages of composting are more reliant on the presence of microbiota within the compost and on their own competitiveness.

### Fungal community composition

Fungi are an important part of the microbiota observed during composting because of their ability to decompose dry recalcitrant acid and low-nitrogen containing substrates in comparison to bacteria [8,11,15].

After data denoising and chimera screening (56 chimera were excluded from the analysis), a total of 61,262 sequences were used for further identification. Approximately 22,284 sequences (36.3%) were identified at the genus level. There were 54 unique OTUs of fungi identified in our research, 45 of which belonged to the *Ascomycota* phylum. The predominance of *Ascomycota* corresponds with the results of other researchers [11,45,74]. Maximum numbers of fungal OTUs were observed by the 30^th^ day of composting with 27 and 24 OTUs in I and IH composts, respectively. Overall, the fungal community was characterized by fewer OTUs and a higher dominance of the main species than the bacterial community.

#### Fungal community composition in the I compost. Initial stage of composting

In the initial stage of composting, *Saccharomycetales* was the dominant order comprising 91.4% of the total population. Three main OTUs from this order played a vital role: *Galactomycesgeotrichum*, *Dipodascusaustraliensis* and *Candida sake* with 85, 4 and 2%, respectively (Table 4).

**Table 4:** The most abundant fungal OTUs found in I and IH composts.

Phylum	Class	Order	The most abundant OTUs/taxa (if applicable)	Relative fungal abundance in composts of different ages, %							
				I compost				IH compost			
				days of composting							
				2	30	150	270	2	30	150	270
*Ascomycota*	*Saccharomycetes*	*Saccharomycetales*	*Galactomyces geotrichum*	84.7	18.7	35.3	3.7	70.1	45.2	4.7	9.0
*-*	*-*	*-*	uncultured unidentified fungus	0.2	65.6	44.1	3.3	0.0	23.3	68.9	40.6
*Ascomycota*	*Sordariomycetes*	*Microascales*	*Pseudallescheria boydii*	0.0	0.9	11.6	38.9	0.0	0.6	11.6	43.8
*Ascomycota*	*Orbiliomycetes*	*Orbiliales*	*Arthrobotrys thaumasia*	0.0	0.0	0.0	50.0	0.0	0.0	0.0	0.0
*-*	*-*	*-*	unclutured soil fungus	8.4	0.3	3.6	0.2	17.4	2.7	0.5	4.5
*Ascomycota*	*Sordariomycetes*	*Microascales*	*Graphium* sp	0.0	0.0	0.0	0.0	0.0	3.8	3.2	0.0
*Basidiomycota*	*Agaricomycetes*	*Agaricales*	*Coprinus cordisporus*	0.0	1.3	0.0	0.0	0.0	14.4	0.0	0.0
*Ascomycota*	*Saccharomycetes*	*Saccharomycetales*	*Dipodascus australiensis*	3.7	0.4	1.7	0.1	8.3	0.2	0.0	1.0
*Ascomycota*	*Saccharomycetes*	*Saccharomycetales*	*Metschnikowia aff*. *fructicola*	0.0	0.0	0.0	0.0	0.0	5.6	0.0	0.0
*Ascomycota*	*Saccharomycetes*	*Saccharomycetales*	*Candida sake*	2.2	0.0	0.0	0.0	2.8	0.0	0.0	0.0

*Saccharomycetales* yeast is associated with the early stages of composting [8,45,74]. *G*. *geotrichum* is described as a very efficient decomposer of organic compounds from easily degradable dissolved organic matter in the wastewater sludge to recalcitrant molecules such as textile dyes [75–77]. It was found to be abundant in the early stages of the composting process by López-González and colleagues (2015) [73]. In addition, *Galactomyce* sp. was observed in composts by Hansgate and colleagues (2005) [49]. *Dipodascus australiensis* was found previously on the first day of composting from the municipal waste compost windrows [33]. *Candida* species are often found in composts [33,49,74]. *Candida sake* is reported to be a human health pathogen associated with endocarditis [78].

Besides *Saccharomycetales*, one fungal OTU (uncultured soil fungus) dominated the initial compost. It was not identified using standard QIIME procedures. Alignment of its sequence with those in the UNITE database [79] allowed us to identify *Galactomyces* sp. with 100% similarity, which is found in rhizospheres [80].

#### Evolution of fungal community in the process of composting

Over time, the abundance of *Saccharomycetales* decreased and was equal to 4% in the final product. In contrast, the abundance of *Microascaceae (Sordariomycetes*, *Ascomycota)* increased continuously from zero in the beginning to 39% at the completion of the process. High percentages were observed for the order *Orbitales (Orbilimycetes*, *Ascomycota)* in the final product (50%) and for uncultured unidentified fungus on the 30^th^ and 150^th^ days (66 and 50%, respectively). Significantly lower abundances (from 1 to 3%) were observed for *Ascomycota Plesporales (Dothideomycetes)*, *Eurotiales (Eurotiomycetes)*, *Sordariales (Sordariomycetes)*, *Hypocreales (Sordariomycetes)* and *Basidiomycota Agaricales (Agaricomycetes)* and uncultured soil fungus that was found on the 2^nd^ day of composting.

All of the dominating taxa and taxa with lower abundances mentioned above were previously identified in composts [8,22,68,73].

Sequences from the uncultured fungi that dominated on the 30^th^ and 150^th^ days of composting with 66% and 41%, respectively, were processed in the UNITE database [79], and there was a 100% similarity with fungi observed in mining waste under remediation [81].

Both the orders that dominated in the fungal community on the 270^th^ day of the process (*Microascaceae* and *Orbitales*) were represented by only one OTU: *Pseudallesheria boydii* with 39% and *Arthrobotrys thaumansia* with 50%, respectively. *Pseudallesheria boydii* was described as a thermo-tolerant strain which develops and becomes abundant during the thermophilic stage of composting [8]. *Arthrobotrys* sp. is a nematophagous fungus observed in composts [11,82], and emergence of nematodes in composts is viewed as one indicator of compost maturity [66].

Thus, fungal community composition and succession reveals that composting of the waste mixture proceeds normally and efficiently.

#### Fungal community composition in the IH compost. Initial stage of composting

During the initial stage, *Saccharomycetales* dominated (82%) with three main OTUs, *Galactomyces geotrichum*, *Dipodascus australiensis* and *Candida sake* in the IH compost, and uncultured soil fungus was the second highest in abundance (17%).

#### Evolution of fungal community in the process of composting

The dynamics of fungal composition in the IH mixture changed resembling that of the I mixture, including a decrease in *Saccharomycetales* and an increase of *Microascaceae* abundances. Several differences were observed in IH and I composts. First, the abundance of *Coprinus cordisporus* from *Agaricales* orders was 11 times higher on the 30^th^ day; in addition to this, the presence of *Metschnikowia aff*. *fructicola* was observed on the 30^th^ day. *Metschnikowia* sp. are usually isolated from terrestrial habitats and associated with flowers or fruits and insect presence [83]. Finally, *Arthrobotrys* sp. was not observed in the final compost, and the presence of uncultured unidentified fungi in it remained high throughout the entire composting process.

In general, the succession of fungal community structures in the two composts investigated was quite similar.

### Pathogenic microbes in the I and IH composts

Composting is reported to be a good tool for eliminating pathogen strains that cannot tolerate the high temperatures of the thermophilic stage [3]. Both compost types were analyzed for the presence of human pathogens of bacterial and fungal origin in all sampling points.

During the first month of composting, pathogenic bacterial OTUs were found in both composts: *Streptococcaceae*, *Staphylococcus*, *Rickettsiales* and *Treponema*. In the final product, *Streptococcaceae* and *Staphylococcus* were not present and the abundance of *Rickettsiales* and *Treponema* decreased and did not exceed 0.7%. Notably, the dynamics of these four strains were similar in both composts. Sequences associated with *Escherichia*, *Listeria or Salmonella* were not found in any of the compost samples.

As reported in the literature, final compost products may cause an opportunistic hazard to human health mainly due to saprotrophic fungi such as *Aspergillus fumigatus*, *Candida krusie*, *Alternaria alternata*, which can potentially cause mycoses of the bone marrow. It is discussed in the literature that the abundance of pathogenic fungi in the final composts is underestimated and requires further examination [22,33,64]. In the composts analyzed in this study, only two OTUs were identified that can be hazardous to humans. The fungal pathogen *Candida sake* was found in both composts during the early stage, but it was later eliminated. *Fusarium* sp. was present in the final composts in minor quantities (up to 0.2%). *Fusarium* sp. can be pathogenic towards humans and plants, however, some species are not pathogenic and these are typically found in composts [22].

### Alpha diversity of bacterial and fungal communities of I and IH composts

For bacterial and fungal communities of I and IH composts, the alpha diversity indices derived from the analysis of 454 pyrosequencing results of the samples collected during composting process are reported in Table 5. By analysis of the indices calculated we took into account the Shannon-Weaver index which is expected to be higher in the community with the highest number of OTUs but of similar frequency. The Simpson index is lower when one OTU predominates, and evenness characterizes equitability of the species within a community with 1 being complete evenness [39,41,84].

**Table 5:** Alpha diversity indices of the fungal and bacterial communities of I and H composts.

kingdom	Index	Alpha biodiversity indices							
		I compost				IH compost			
		days of composting							
		2	30	150	270	2	30	150	270
Bacteria	Shannon-Weaver	1.55	3.26	2.99	2.85	3.27	3.85	3.81	4.19
	Simpson	0.68	0.89	0.87	0.86	0.87	0.96	0.94	0.97
	Evenness	0.4	0.67	0.65	0.6	0.65	0.8	0.76	0.82
Fungi	Shannon-Weaver	0.66	1.29	1.27	1.14	0.91	1.67	1.01	1.19
	Simpson	0.29	0.53	0.65	0.6	0.46	0.73	0.49	0.63
	Evenness	0.25	0.39	0.48	0.49	0.41	0.52	0.44	0.48

Three general conclusions can be drawn concerning indices. First, values calculated for the 2^nd^ day of composting differed significantly from those of the other sampling time periods, for all three indices for both bacterial and fungal communities. Second, the values calculated for IH composts were higher in most cases compared to those of I composts, especially on the 2^nd^ day. Third, within one kingdom (bacteria or fungi), the indices fluctuated in relation to each other; therefore, for bacteria the correlation coefficient for the pairs of indices was equal to 0.99, and for fungi it ranged between 0.79 and 0.94. A low correlation was found between fungal and bacterial communities for one and the same index values.

In I composts, the lowest values for diversity indices in both fungal and bacterial communities were obtained on the 2^nd^ day. They were 1.6–2.2 folds lower than the corresponding maximum values. Undeniably, the initial communities comprised a few very dominant OTUs. These OTUs originated from the raw wastes where most likely inhibiting factors such as heavy metals, oily compounds and/or high humidity prevented high biodiversity by the selection of strains tolerant to unfavorable environmental conditions. The diversity indices rose by 1.3–2.1 folds until day 30 when the values for all bacterial indices and the fungal Shannon-Weaver index were the highest. This indicates that (1) microbial communities go through serious successions within 28 days, which are made possible most likely due to the artificial establishment of optimal nutrient and moisture content and aeration; (2) fungal and bacterial communities do not change simultaneously, probably due to different growth speeds and level of recalcitrance of preferred substrates; and (3) compost on the 30^th^ day is transitioning from thermophilic to cooling stages, and therefore still contains thermophilic and already contains mesothermic OTUs. The diversity indices of compost on the 150^th^ and 270^th^ days remained high indicating that conditions were optimal, and communities had stabilized.

In IH composts, the diversity indices calculated for the 2^nd^ day are significantly higher than those in H composts. This may be explained by the compost mixture composition of the two composts. For example, the SS used in the IH composts contained less heavy-metals meaning that it was less toxic, and therefore microbial diversity was higher. In addition, the IH compost contained less oily polluted sawdust which could be a source of oil-degrading microbes, leading to a reduction in diversity. In terms of maximal diversity index values, the bacterial and fungal communities of the IH compost differed. For fungal communities, maximal indices were obtained on the 30^th^ day of composting for similar reasons as described above. In contrast, in all bacterial communities, alpha diversity indices rose from the 2^nd^ to the 270^th^ day meaning that the IH compost became more optimal for bacterial populations with time, and new niches had formed. This difference in trends once more supports the idea that bacterial and fungal composting communities do not develop simultaneously.

### Beta diversity of bacterial and fungal communities of the I and IH composts

**Fig 2:**
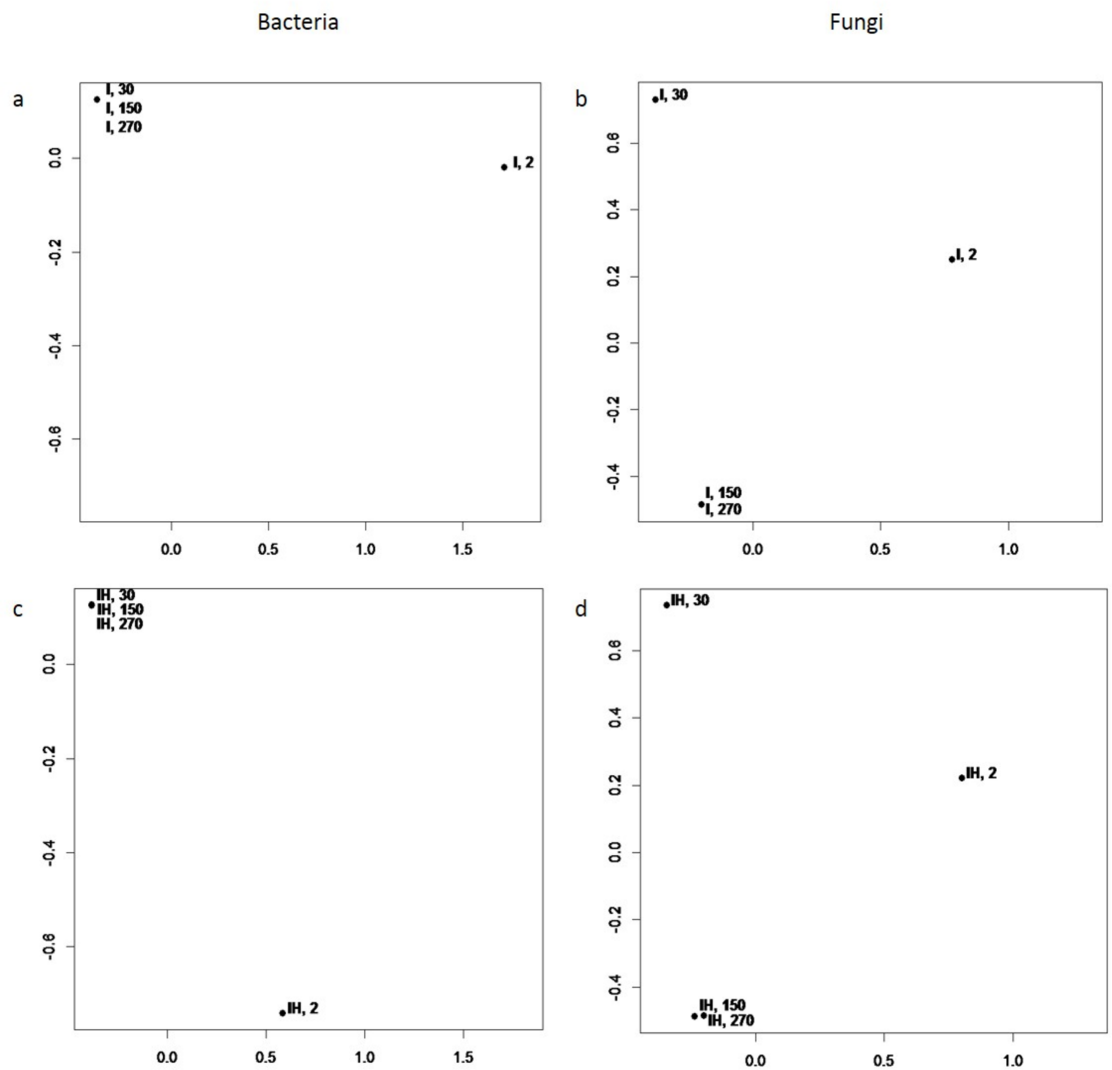
NMDS plots of microbial communities of I and IH composts. a–bacterial communities of the I composts; b–fungal communities of the I composts, c–bacterial communities of the IH composts; d–fungal communities of the IH composts, sampled on the 2^nd^, 30^th^, 150^th^ and 270^th^ day of incubation.

Beta biodiversity characterizes similarities and dissimilarities between different communities [85]. NMDS is a commonly used ordination method used for ecological community data [42,43]. In Fig 2A–2D, NMDS plots are demonstrated, where points represent microbial communities; the closer the points are situated, the more similar are the communities.

Bacterial communities of the I compost sampled on the 30^th^, 150^th^ and 270^th^ days are close to each other in terms of their species composition, and the I compost sampled on the 2^nd^ day differs significantly from these three (Fig 2A). Similarly, the bacterial community of IH composts sampled on the 2^nd^ day differs from those in composts subsequently sampled (Fig 2C). Therefore, the bacterial succession is significant within the first month of composting, and later bacterial communities remain stable. Notably, this process was found to be identical in two different composts.

Fungal succession to the stable community of mature composts was slower. As follows from Fig 2B, compositions of the fungal community on the 150^th^ and 270^th^ days were close, while compositions of the 2^nd^ and the 30^th^ days differed from each other as well as from the subsequent days. Again, the trend described was similar in both composts (Fig 2B and 2D), while it differed from the bacterial succession pattern. This finding is consistent with that of the alpha biodiversity analyses described above.

Significant differences between the communities residing in the initial and final composts support our idea about the absence of recolonization of the mature compost by the microbes of the mesophilic stage. In addition, the results of NMDS plotting demonstrate that the nature of the community successions is similar in different composts. We suggest that such successions may lead to the formation of similar communities in the final composts of different origins due to the presence of similar microbial species and identical physical conditions of composting. To address this assumption, that communities’ dissimilarity tends to decrease with time, we estimated the tendency significance. A linear model of the form $E\left[diss_{T_{1},T_{2}}\right]=a_{0}+a_{1}T_{1}+a_{2}T_{2}$ was fitted, where $diss_{T_{1},T_{2}}$ is the dissimilarity between the communities at time T1 and T2. Coefficients a1, a2 are negative and have p-values < 0.01 for both the bacterial and fungal communities. Thus, it was shown that over time the similarity of bacterial communities of the two composts increased, and in the final product communities became more similar than in the beginning of composting. The same trend was observed for the fungal community.

## Conclusion

The results indicate that the microbial composition of the I and IH composts depended on the raw wastes used to develop the compost mixtures, on microbial competitiveness and on the duration of composting; however, the significance of these factors varied at different sampling times. The initial composition of both bacterial and fungal communities differed significantly from the subsequent communities as revealed by changes of the dominants present. No re-colonization of the composts by the species present in the mesophilic but absent in the thermophilic stage was observed. The dynamics of the bacterial and fungal communities between the two composts was similar. In contrast, bacterial and fungal communities within the same compost did not change correspondingly. Bacterial and fungal communities in the two composts became more similar in the final than the initial composts and those sampled on the 150^th^ and 270^th^ days.

## Supporting information

S1 Table Abundance of bacterial OTUs in the I and IH composts on the 2nd day of composting. (XLS)

S2 Table Abundance of fungal OTUs in the I and IH composts on the 2nd day of composting. (XLS)

S3 Table Correlation coefficients between abundances of bacterial and fungal OTUs in three replications of the compost I. (DOCX)

S4 Table Correlation coefficients between abundances of bacterial and fungal OTUs in three replications of the compost IH. (DOCX)

S5 Table Chi-square p-values for preliminary experiments data. (DOCX)

S1 Text Design of the preliminary experiments. (DOCX)
