# Supplementary material for: Fungal and bacterial successions in the process of co-composting of organic wastes as revealed by 454 pyrosequencing
Source: PLoS One. 2017 Oct 23;12(10):e0186051. doi: 10.1371/journal.pone.0186051 (PMC5653195; doi:10.1371/journal.pone.0186051)
Supplement: S3 Table — (DOCX) [file pone.0186051.s003.docx]

S3 Table. Correlation coefficients between abundances of bacterial and fungal OTUs in three replications of the compost I

| Bacteria | mixed sample | replicates | | |
| --- | --- | --- | --- | --- |
|  | I, 2 day | I, 2a | I, 2b | I, 2c |
| I, 2 day | 1.0000000 | 0.9998413 | 0.9982814 | 0.9992833 |
| I, 2a | 0.9998413 | 1.0000000 | 0.9990623 | 0.9985074 |
| I, 2b | 0.9982814 | 0.9990623 | 1.0000000 | 0.9953742 |
| I, 2c | 0.9992833 | 0.9985074 | 0.9953742 | 1.0000000 |
| Fungi | I, 2 day | I, 2a | I, 2b | I, 2c |
| I, 2 day | 1.0000000 | 0.9999588 | 0.9999916 | 0.9999863 |
| I, 2a | 0.9999588 | 1.0000000 | 0.9999453 | 0.9999380 |
| I, 2b | 0.9999916 | 0.9999453 | 1.0000000 | 0.9999980 |
| I, 2c | 0.9999863 | 0.9999380 | 0.9999980 | 1.0000000 |
