# Supplementary material for: Fungal and bacterial successions in the process of co-composting of organic wastes as revealed by 454 pyrosequencing
Source: PLoS One. 2017 Oct 23;12(10):e0186051. doi: 10.1371/journal.pone.0186051 (PMC5653195; doi:10.1371/journal.pone.0186051)
Supplement: S4 Table — (DOCX) [file pone.0186051.s004.docx]

S4 Table. Correlation coefficients between abundances of bacterial and fungal OTUs in three replications of the compost IH

| Bacteria | mixed sample | replicates | | |
| --- | --- | --- | --- | --- |
|  | IH, 2 day | IH, 2a | IH, 2b | IH, 2c |
| IH, 2 day | 1.0000000 | 0.9996409 | 0.9991979 | 0.9997184 |
| IH, 2a | 0.9996409 | 1.0000000 | 0.9995449 | 0.9995044 |
| IH, 2b | 0.9991979 | 0.9995449 | 1.0000000 | 0.9988992 |
| IH, 2c | 0.9997184 | 0.9995044 | 0.9988992 | 1.0000000 |
| Fungi | IH, 2 day | IH, 2a | IH, 2b | IH, 2c |
| IH, 2 day | 1.0000000 | 0.9999833 | 0.9998211 | 0.9999973 |
| IH, 2a | 0.9999833 | 1.0000000 | 0.9997081 | 0.9999821 |
| IH, 2b | 0.9998211 | 0.9997081 | 1.0000000 | 0.9998118 |
| IH, 2c | 0.9999973 | 0.9999821 | 0.9998118 | 1.0000000 |
