# Supplementary material for: Fungal and bacterial successions in the process of co-composting of organic wastes as revealed by 454 pyrosequencing
Source: PLoS One. 2017 Oct 23;12(10):e0186051. doi: 10.1371/journal.pone.0186051 (PMC5653195; doi:10.1371/journal.pone.0186051)
Supplement: S5 Table — (DOCX) [file pone.0186051.s005.docx]

S5 Table. Chi-square p-values for preliminary experiments data

|  | I, 2 | IH, 2 |
| --- | --- | --- |
| Bacteria | 0.9999963 | 0.9481447 |
| Fungi | 0.9922934 | 0.9843558 |
