# Supplementary material for: Fungal and bacterial successions in the process of co-composting of organic wastes as revealed by 454 pyrosequencing
Source: PLoS One. 2017 Oct 23;12(10):e0186051. doi: 10.1371/journal.pone.0186051 (PMC5653195; doi:10.1371/journal.pone.0186051)
Supplement: S1 Text — (DOCX) [file pone.0186051.s006.docx]

To assess the similarity of replications, we conducted preliminary experiments for day 2 for both bacteria and fungi in three replicates. DNA was extracted from three samples sampled independently and sequenced using 454-pyrosequencing method. The results were processed using QIIME 1.6.0 program (Caporaso et al., 2010), and statistical analysis was done using R Statistical Software (R 3.0.0) package (R Development Core Team, 2013). Besides, DNA from the three independent samples was mixed in a ratio 1:1:1 and treated in the similar way. The community composition expressed on the OTU level is presented in S1 Table for bacteria and S2 Table for fungi.

For the results presented in S1 and S2 Tables, the correlation coefficient was calculated. As shown in S3 and S4 Tables, it ranged between 0.9953742 and 0.9998413 for bacteria, and between 0.9998118 and 0.9999980 for fungi. That means that the results obtained for 3 independent and one mixed samples highly correlated between each other.

Besides, replicates were used to estimate errors distribution assuming independent errors for different taxa, and mixed sample was compared with the mean of replicates. For this purpose, Chi-squared criteria was used. It was revealed that all the Chi-square p-values were greater than 0.05 (S5 Table) which means that the mixed sample was statistically not different from the independent ones.

Summarizing the statistical analysis of the community structure of the two composts sampled on the day 2, we concluded that that the mixed samples’ analysis results may substitute separate analysis results of the independent samples.

REFERENCES

Caporaso JG, Kuczynski J, Stombaugh J, Bittinger K, Bushman FD, Costello EK, et al. QIIME allows analysis of high-throughput community sequencing data. Nat Methods. 2010;7: 335–336.

R Development Core Team: R: A language and environment for statistical computing, R Foundation for Statistical Computing, Vienna, Austria, 2013.
